# Impact of Paternal Postpartum Depression on Maternal and Infant Health: A Narrative Review of the Literature

**DOI:** 10.7759/cureus.66478

**Published:** 2024-08-08

**Authors:** Hussein Attia Hussein Mahmoud, Mohit Lakkimsetti, Maria Jimena Barroso Alverde, Pranav S Shukla, Alviya T Nazeer, Sukesh Shah, Yuktha Chougule, Amisha Nimawat, Swetapadma Pradhan

**Affiliations:** 1 Diagnostic Radiology, Heliopolis Hospital, Cairo, EGY; 2 Internal Medicine, Mamata Medical College, Khammam, IND; 3 Internal Medicine, Anahuac University, Mexico City, MEX; 4 Medical School, Grant Medical College and Sir J.J. Group of Hospitals, Mumbai, IND; 5 Obstetrics and Gynaecology, Government Medical College and Hospital, Pudukkottai, IND; 6 Medical School, American University of Integrative Sciences, Bridgetown, BRB; 7 Medical School, Kazan State Medical University, Kazan, RUS; 8 Internal Medicine, Interfaith Medical Center, New York, USA; 9 Medical School, European University Faculty of Medicine, Tbilisi, GEO

**Keywords:** child development, major depressive disorder, postpartum depression, maternal-child health, paternal postpartum depression

## Abstract

Postpartum depression (PPD) has been widely studied, assessed, and promptly intervened in new mothers. However, paternal postpartum depression gained attention not long ago. Postpartum depression in men could present over one year following the birth of the child, frequently presenting with symptoms like irritability, low mood, sleep disturbances, changes in appetite, fatigue, and loss of interest in everyday activities; amongst other symptoms of Major Depressive Disorder which may hinder them from taking care of themselves and the baby. Paternal PPD significantly impacts partner relationships causing maternal PPD, poor infant bonding, and therefore, affecting overall child development. The following narrative review is based on a literature search of articles published on paternal postnatal depression. The primary emphasis of this review has been to provide an overview of the current comprehension of paternal postpartum depression regarding prevalence, global incidence, and risk factors and to explore potential diagnostic tools for assessment and interventional strategies to treat this condition. Interestingly, pandemic-related stressors have been positively attributed to an increase in PPD prevalence post-pandemic. While more research is being conducted on this subject, research on the measurement characteristics of the diagnostic tools is highly recommended to implement well-defined criteria for early diagnosis of paternal PPD. The significant adverse consequences of PPD for not just the new mother, but also the infants, necessitate proper and timely diagnosis of PPD. Despite its severity, there have been no specific treatment modalities.

## Introduction and background

Pregnancy carries a wavelength of emotions, ranging from excitement, happiness, and awe-inspiring to anxiety, being overwhelmed, and vulnerability. These emotions keep fluctuating throughout the pregnancy and are mostly attributed to the hormonal changes a woman goes through while being pregnant. However, these hormonal changes affect the mental status of the woman not just during pregnancy, but also after giving birth. These strong feelings/emotions are often referred to as ‘baby blues’ if they last for a few days up to 2 weeks and encompass a variety of emotions like anxiety, crying spells, mood swings, sadness, guilt, irritability, feeling overwhelmed, etc. [1, 2]. According to The Diagnostic and Statistical Manual of Mental Disorders 5th Edition (DSM-5), postpartum depression (PPD) and major depressive disorder (MDD) have been assigned the same diagnostic criteria. This type of depression usually occurs within 1-3 months post-delivery but can also happen up to 12 months postpartum. The individuals experiencing this typically also have sleep disturbances, appetite changes leading to weight loss/gain, fatigue, suicidal ideation (about oneself or the infant), and anhedonia which may hinder them from taking care of the baby or themselves [1, 2].

The average rate of prevalence of PPD in Western countries is believed to be 10-15% [3]. Another review that involved 143 studies from 40 countries identified an extensive range of PPD prevalence rates, ranging from 0.5% to around 60% [4]. The speculated reason behind such a wide range is believed to be multifactorial including but not limited to, socio-economic status (financial status, social support, nutrition, safe households), unwanted pregnancies, cultural beliefs and the stigma surrounding mental health, and different ways of reporting. A recent meta-analysis showed that there were statistical differences in the prevalence between different geographical regions, with the Middle East having the highest prevalence (26%, 95% CI 0.13-0.39) and Europe having the lowest (8%, 95% CI 0.05-0.11) [5]. Moreover, the prevalence of PPD in low-middle-income countries is the highest (20.14 (range: 16.39 - 24.50)) in comparison with upper-middle and high-income countries [6]. 

There isn't one trigger that leads to the development of PPD; instead, it’s a combination of multiple aspects of the mother’s life before, during, and after pregnancy. A woman goes through a myriad of endocrine changes throughout and after pregnancy. During pregnancy, there is an elevation of progesterone and estrogen levels, among other hormones; while after giving birth, there is a steep fall in those levels [7]. The sudden, steep fall in those hormones affects the brain chemistry, which manifests as mood swings, anxiety, sleep disturbances, and emotional lability seen during the postpartum period. There are several risk factors, however, that are believed to contribute to the development of PPD. A history of substance abuse, a stressful life event like losing a job, losing a family member or a friend, inadequate spousal/family support, any medical complications before or during pregnancy, as well as complications during birth [7].

The significant adverse consequences of PPD for not just the new mother, but also the infants, and other members of the family necessitate proper and timely diagnosis of PPD. The most common tool used to screen for PPD is the EPDS (Edinburgh Postnatal Depression Scale). It is a self-report questionnaire consisting of 10 items ranging from 0-3 about how the pregnant/postpartum individual has experienced the past week. The numerical score against each item is then added to obtain a total score for the patient. Another common way to screen is the PHQ-9 (Patient Health Questionnaire), which traces the patient’s experiences and feelings in the past two weeks. The desirable time for these screening tools to be implicated is the first postnatal visit to the doctor since the symptoms of PPD begin around that time in the postpartum period [8].

**Figure 1:**
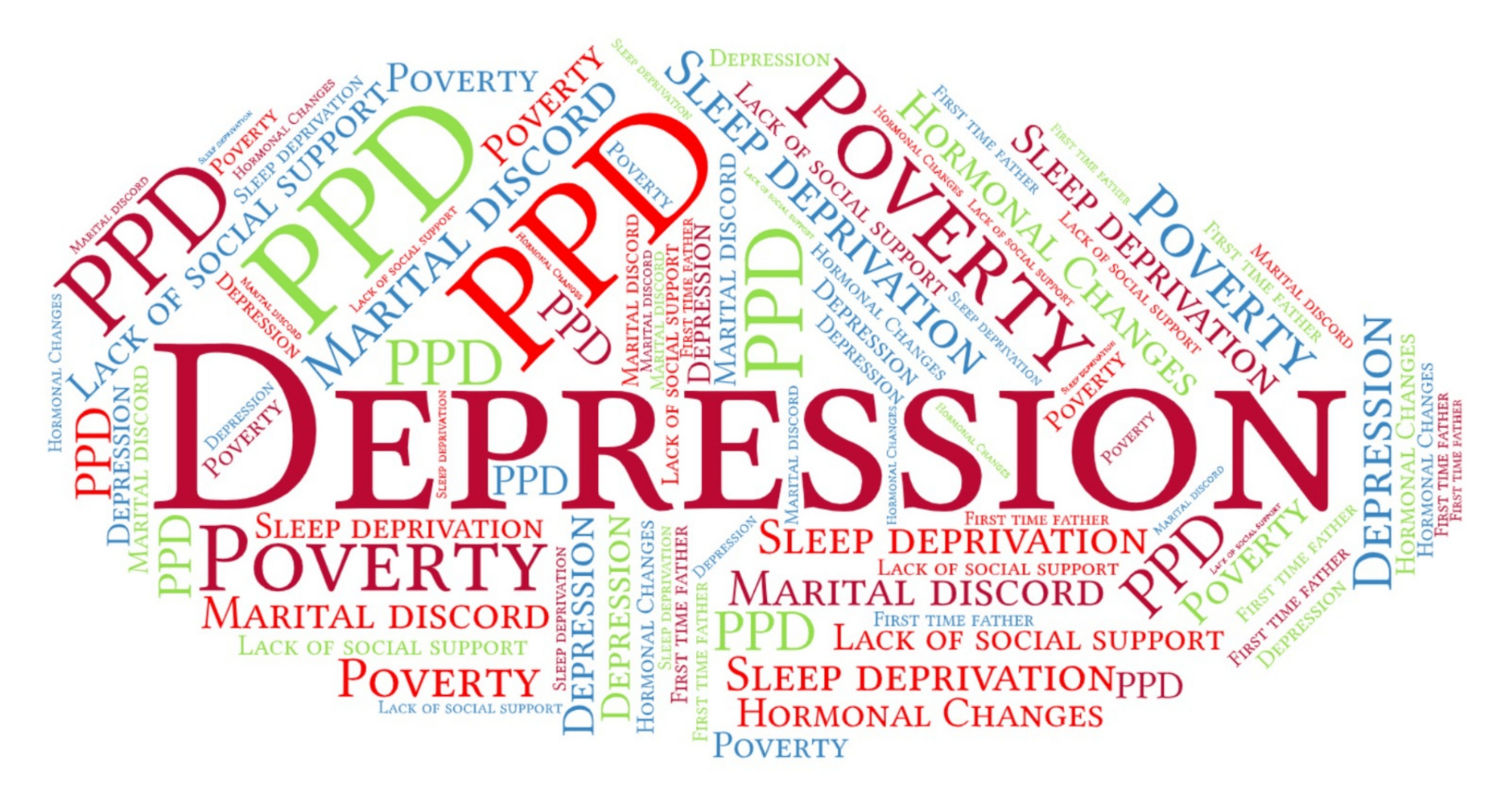
Word cloud of key terms for postpartum depression.

While it is relatively common in women it can also be seen in men. PPD in men could present over one year following the birth of the child, frequently presenting with symptoms like irritability, low mood, and loss of interest in everyday activities, amongst other symptoms of MDD [9]. A positive psychiatric history (especially depression) in either parent, poverty, and hormonal changes are recognized risk factors [9]. The outcomes further suggested that marital relationship satisfaction and psychiatric history in either or both partners were risk factors most significantly associated with the development of PPD in men [10]. Furthermore, evidence suggests a relationship between paternal employment, psychological status, history of maternal mental illness, first pregnancy, and paternal PPD [11]. In 2007 Kim and Swain published a paper that sheds light on biological and ecological risk factors involved in the etiology of paternal PPD. Their study discusses how hormonal imbalances such as low levels of testosterone, estrogen, cortisol, oxytocin/vasopressin, and prolactin as biological risk factors, in addition to ecological risk factors like marital life problems and maternal/paternal mental health history can modulate paternal PPD [12]. Figure 1 shows a word cloud comprising the different keywords for paternal postpartum depression.** **Various relevant keywords were collected during our literature review from Google Scholar and PubMed to compose this word cloud.

In 2015, Nishimura et al. conducted a study that reported the prevalence of paternal depression to be 13.6% in Japan at the 4-month postpartum period [9]. Other studies suggested the prevalence of paternal PPD to be up to 25.5% (range 1.5%-25.5%) [11]. In 2017, Wang D. et al. conducted a study that showed that both the PHQ-9 and the EPDS were equally efficacious while screening for major depressive episodes [10]. A diagnosis of paternal PPD may often be missed due to fathers not being actively involved in the maternal-child healthcare system. The EPDS - Partner variant, which the mother rates, was found to be a valid, reliable measure of paternal depression when compared to other well-validated screening methods [13].

The EDPS scale consists of 10 self-report answers, eight of which deal with symptoms of depression like sadness and self-blame. The remaining two address symptoms of anxiety like experiencing excess worry or fear and feeling scared or panicky [14]. Patient response is scored on a scale of 0-3 based on the severity of symptoms. Cut-off scores for a diagnosis of PPD vary from 9-13 points out of a maximum score of 30 [15]. The EDPS scale has been validated for a diagnosis of PPD in men as well as women [16]. Also of note, is a 2009 paper by Mitchell AJ suggesting that the shorter three-item sub-scaled EDPS may be as effective as the 10-item questionnaire [17]. Other screening methods used as self-reported questionnaires that may be used to evaluate for PPD include the Beck Depression Inventory (BDI), the General Health Questionnaire (GHQ), and the Centre for Epidemiological Studies - Depression (CES-D) scale [5]. The CES-D scale is a 20-item questionnaire. Scores greater than or equal to 16 out of 20 indicate depression, based on patients' symptoms over the past 7 days [9]. The CES-D scale can be used to screen for depression in culturally diverse populations and has a high specificity and sensitivity [8].

The PHQ-9 records patient responses at four levels ranging from “not at all” to “nearly every day”, used to assess how the patient has felt in the past 2 weeks [9]. Boyd et al. in their comprehensive review of screening instruments identified the GHQ and the BDI I or II as additional instruments that can be used to assess perinatal symptoms of depression [18].

## Review

Global incidence of paternal postpartum depression

Compared to fathers of term babies, fathers of preterm infants exhibit more postnatal depression symptomatology. Early cognitive development in children is also significantly impacted by the depressed symptoms of fathers [19] since fathers have dual responsibilities in the early hours and days following the delivery. Providing post-partum care for their partners and serving as the child’s main caregiver. Most continue to assist with their infant's daily care during the hospital stay and beyond, frequently balancing employment and other obligations. Having a preterm baby adds to anxiety, especially for those fathers who are not aware of the problem and its management [20]. Parents of preterm children are at higher risk of postpartum depression at 9 months postpartum [21]. Compared to parents of full-term babies, fathers of preterm infants had a higher rate of depression. The ratio is 5% vs 36% at 6 months 6% vs 19% and anxiety at birth 10% vs 47% and at 6 months 10% vs 20%, respectively [20]. Out of families reporting preterm birth 8.9% of fathers showed signs of postpartum depression at around 9 months after birth. Comparing fathers of extremely and moderately pre-term newborns to fathers of full-term babies, studies show a doubling in the risk of PPD, which is statistically significant [21].

It seems that many parents endure chronic distress and may require further “psychological care”, both during and after pregnancy. Around 4% of children born have a genetic or physical anomaly. It must be traumatic for parents to learn their expectant child has a predicament. When parents decide not to end their pregnancy, their stress levels stay high for the whole pregnancy and often even after the baby is born [22]. A meta-analysis in 2010 estimated the paternal PPD rate as 10%; however, in the Middle East and Saudi Arabia, PPD is a new concept. Egypt (Table 1) has reported a 31.8% prevalence in postpartum fathers and Canada 13.3% [23]. Previous reports have shown that chronically ill children with congenital anomalies are psychosocial burdens and parents have poor quality of life [24-28]. Parents of children with critical congenital heart defects are at a higher risk for mental health morbidity. Putting up with the children’s increased risk of doctor’s visits and procedures, visiting the doctor often, different nutrition patterns in these children, and illness results in significant financial, emotional, and family costs [27].

**Table 1:** Prevalence of paternal postpartum depression in Egypt and Canada.

#	Location	Prevalence
1	Egypt	31.8%
2	Canada	13.3%

Fathers who have unwanted/unintentional pregnancies have a measurable increased risk of developing postpartum mental issues. About 48% of pregnancies around the world happen unplanned [29]. Available data from online discussion forums for males indicates a forum between poor postpartum mental health and unexpected fatherhood. Results from various studies have indicated that fathers who became pregnant unintentionally had worsening mental health within the first year after giving birth and are more likely to have signs of depression during this time. Fathers may also develop post-traumatic stress disorder (PTSD) not only in the immediate time but also up to a year after birth [30]. Since the desire for children is not universal, it is normal for fathers with unintended pregnancies to feel overwhelmed. Some were concerned that not wanting children would be seen as abnormal. A few expressed feelings of regret, sadness, and guilt, some felt like the Tin Man without a heart, while others had no attachment to the baby. Many felt distressed and had a feeling of loss, about their previous life. Not every man was in pain; some were sad, hopeful, excited, terrified, and freaking out while feeling happy [30]. After the birth of a child, fathers must receive support, but the family and health system focus primarily on mothers and babies. It is equally important to be better equipped and trained to identify fathers at risk of mental problems [30]. The study suggests men whose partners conceived unintentionally had twice the risk of developing PPD [29]. Research published in a journal of affective disorders involving around 8000 fathers in total from 23 different studies in many countries including the UK, Canada (Table 1), Japan, China, and the USA suggested mental health outcomes due to unwanted pregnancies are worse in economically weaker countries. Despite widely held beliefs, fathers of numerous children or first-time fathers have similar risks when it comes to having an unplanned child. Around 40% of pregnancies in Australia are unplanned, untimed, or unwanted [29].

Fathers should not be disregarded following the delivery of preterm children. Fathers have significant rates of anxiety and depression on par with what mothers have. Fewer people are aware of the difficulties fathers endure, and as a result, they receive limited support [20]. Some fathers might experience hormonal changes during and after birth. The hormone change is thought to assist in having a strong father-child relationship [31]. Decreased testosterone and increased estrogen in new fathers have been recorded. An increase in other hormones, e.g. cortisol, prolactin, and vasopressin, has been observed. However, this hormonal change can lead to PPD or exacerbate the existing symptoms. The lower level of testosterone causes depression in men, whereas a low level of other mentioned hormones can lead to attachment problems between the father and his newborn, which can cause a mood disorder [31]. 

Tools for recognizing pregnancy-related criteria for paternal depression

Mothers go through a lot of psychological adjustments during the postpartum phase, which increases their risk of depression. This is also true for fathers. Therefore, it is notably critical to examine the validity of these measures for males and create precise diagnostic instruments to aid in the diagnosis of paternal PPD [12]. We have mentioned (Table 2) a few of the significant scales for evaluating the symptoms of fatherhood-related PPD [32]. The EPDS, the CES-D, and the BDI were the three most thoroughly studied measures [32]. Brief Symptom Inventory (BSI), Chinese Health Questionnaire (CHQ), Depression Anxiety and Stress Scale (DASS), Gotland Male Depression Scale (GMDS), Hospital Anxiety and Depression Scale (HADS), Kessler Psychological Distress Scale (K10), Paternal Adjustment and Paternal Attitudes Questionnaire (PAPA), PHQ, Postpartum Depression Screening (PDSS), and Zung's Self-rating Depression Scale (SDS) were among the other 10 instruments that were evaluated in previous studies as well [32].

**Table 2:** Some tools utilized in diagnosing pregnancy-related paternal postpartum depression.

#	Tools
1	The Edinburg Postnatal Depression Scale (EDPS)
2	Centre for Epidemiologic Studies Depression Scale (CES-D)
3	Beck Depression Inventory (BDI)
4	Brief Symptom Inventory (BSI)
5	Gotland Male Depression Scale (GMDS)
6	Depression Anxiety and Stress Scale (DASS)

The Edinburgh Postnatal Depression Scale (EPDS)

The EPDS is a 5-minute quick Multiple Choice Questions (MCQ) test comprising a 10-item short self-reported questionnaire [33]. The patient chooses an answer from a list of available choices that best correlates with how he/she felt during the past week. Scores are calculated based on the responses associated with the symptoms' seriousness. Items 3, 2, 1, and 0 have reversal scores, as do items 5 through 10. Scores above 12 or 13 likely show PPD and should be followed by clinical evaluation for further confirmation of perinatal depression. It is advisable to repeat the test after two weeks.

Center for Epidemiologic Studies Depression Scale (CES-D)

A CES-D [34] is a brief self-report measure created to gauge the general population's level of depression symptomatology [35]. The score is calculated from a sum of the 20 questions. The possible range is 0-60. A score of 16 points or more in this questionnaire is considered PPD. The scale has been found reliable (Alpha>0.85) in previous research [36].

Beck Depression Inventory (BDI)

BDI-IIis a 21-item questionnaire, each representing a depressive symptom. The items are categorized into four groups [37] (somatic/physical, affective/emotional, cognitive, and vegetative symptoms that refer to sleep and appetite). This inventory shows high internal consistency and validity in recognizing depressed subjects [38]. However, limitations are noted being a static tool that is insensitive to symptomatic change over time [39].

Brief Symptom Inventory (BSI)

BSI is a measure of the 53 items that make up the nine main aspects of mental symptoms [40]. In addition to three global indices of distress - the Global Severity Index, the Positive Symptom Distress Index, and the Positive Symptom Total. The following symptoms are also present: obsession-compulsion, interpersonal sensitivity, hostility, depression, anxiety, somatization, phobic anxiety, paranoid ideation, and psychoticism.

Gotland Male Depression Scale (GMDS)

GMDS is a screening inventory made up of a series of 13 short self-assessing scales [41] developed for better detecting male depression. Items on anger, irritation, acting out, and alcohol misuse are included in the questionnaire.

Depression Anxiety and Stress Scale (DASS)

The 42-item DASS assesses the general population's emotional condition in terms of stress, anxiety, and depression [42]. A study was made in 2019 for fathers and mothers who were self-screened using the Edinburgh Postnatal Depression Scale to start getting care as soon as possible [43]. The purpose of this research was to evaluate a referral pathway brochure and self-screening tool for expecting mothers and their partners. Our study design was a single blinded randomized controlled one. Seventy dads in the sample were randomly assigned to receive either standard treatment or the self-screening questionnaire and referral system handout intervention. The EPDS and Kessler Psychological Distress (Kessler-10) are examples of the self-screening tools used. Results showed that fathers consistently reported less emotional anguish than mothers, with disparities between the genders on the EPDS and Kessler-10 scales measured in differences of 1.0 points on the EPDS and 1.0 points on the Kessler-10 [43].

Another search was made in 2022 using 17 studies that met the inclusion criteria and showed that the 10-item Kessler-10 and the BDI both had summary receiver operating characteristic (sROC) curves of 0.91, which was comparable to the EPDS's 0.90 and 0.87 [44]. However, the EPDS's sROC curve was 0.54 when compared to the PDSS's (0.98) [44]. More research on the measurement characteristics of EPDS and other equipment is highly recommended [32] to prompt a better understanding to implement thorough criteria for early diagnosis of paternal PPD.

Criteria/scales we use to measure and screen paternal involvement with a child

Questionnaires can help assess the general condition of the patient and their response to their environment. One 26-item questionnaire known as the responsibility attitude scale (RAS) was assessed in the year 2000 for the correlation of cognition of responsibility to obsessional, anxiety, or depressive symptoms [45]. The responsibility is regarding not the patient’s self, but their offspring. The RAS questionnaire has been adopted into the Parental Responsibility Scale (PRS) which asks the questions from a standpoint of promoting growth and preventing negative harm from befalling the patient’s child or children. Questions were modified, some deleted, and seven additional questions regarding direct concerns of responsibility for a child were added, so the PRS is a 30-item questionnaire. The PRS is tested by the patient answering a numeric value between 1 and 7, 7 being highly agreed with and 1 being highly disagreed with. This test was originally tested with 208 mothers, predominantly British [46].

A large majority of child involvement and PPD scales are geared toward women, leaving the toolset for assessing paternal PPD very slim. Singley et al, in 2018, published a paper regarding the development of an instrument geared towards estimating how a father is present and involved with their infant. The five key underlying metrics to this theorized scale were: warmth, attunement, positive engagement, frustration, and indirect care. Ultimately this gave birth to the Paternal Involvement with Infants Scale (PIWIS) [47]. The PIWIS scale is a 35-item questionnaire that asks fathers questions ranging from their financial contribution to their active involvement in daily parenting for their infant. To test the PIWIS scale, the trial consisted of 456 fathers (64% white, 50% married) to complete the scale. Subsequently, exploratory analyses were performed on the sampled data. They found that, when observing social outcomes such as engagement with the father, self-sufficiency of the infant, and parental and overall life satisfaction, there were statistically significant positive correlations [47].

Another measure for father involvement was developed in 2018, known as the Father Involvement in Health - Preschool (FIH-PS). It was initially generated by conducting cognitive interviews of parents with children in preschool, whilst incorporating existing quality literature. The four primary factors that make up the FIH-PS include general well-being, emotional health, role modeling, and acute illness. The authors of the FIH-PS then asked 560 fathers of preschool children to answer the newly formed scale, which is a 20-item questionnaire today. When conducting extrapolatory analyses on the resulting data, the FIH-PS was found to adequately identify fathers with potentially low involvement with their children [48]. This was performed by taking a statistical average of paternal involvement from the questionnaire and then examining the standard deviations. 

In a paper published in 2018, Pinto et al, discuss the adjustment faced by parents newly transitioning to parenthood, namely Paternal Adjustment and Paternal Attitudes (PAPA). The duration from the second trimester to 6 months postpartum is crucial for this transition to ensure adequate parenting of the offspring. This PAPA ultimately will give insight into this transitional period where the shift to parenthood occurs. This scale was formed with the help of 127 fathers and resulted in the formation of two subscales: PAPA-AN and PAPA-PN, corresponding to antenatal and postnatal, respectively [49]. Pinto et al went on to utilize it in patients and couples’ post-infertility treatment [50].

The goal of the above numerous paternal scales is to understand the paternal gender role better and evaluate ways of better catering to fathers and their attention. A meta-analysis conducted in 2016 with over 41,000 participants estimates that the overall general prevalence of prenatal and postpartum paternal depression is around 8% [51]. In the end, ensuring better mental health for fathers in this fragile period of early infancy will promote a more healthy and holistic early childhood for their infants. More support groups for this sake are being formed [52]. It has been accepted that a positive presence and influence of the father on the child is a beneficial event [53].

Effect of paternal postpartum depression on mothers and infants

Postpartum depression, a growing mental health concern, has been predominantly associated with mothers. However, the nurturing and responsiveness of parents during a child's initial year can leave lasting impacts on their growth [54]. This highlights the significance of PPD not only on fathers but also on family members, including mothers and infants. Furthermore, there is a notable correlation between the incidence of paternal PPD and maternal PPD [55]. A father suffering from PPD often withdraws from family relationships, increasing the mother’s sense of isolation and inability to rely on her partner for assistance with the infant and household tasks. This dynamic can be a risk factor for maternal PPD [55]. Fathers with PPD exhibit negative behaviors like decreased positive emotions, warmth, sensitivity, irritability, indecisiveness, and increased hostility, intrusiveness, and disengagement. These behaviors can escalate to illicit drug use, marital conflict, and even partner violence [56], further contributing to maternal depression.

When both parents are depressed, negative parenting outcomes are amplified. Research indicates that children of fathers experiencing PPD are more likely to exhibit behavioral issues, particularly between the ages of 3 and 5 years [54]. Depressed parents are more likely to perceive their children negatively and fail to provide adequate care. For instance, studies have shown that depressed fathers are more likely to spank their children [55], highlighting the cycle of negative impacts stemming from paternal PPD. Paternal PPD is characterized by reduced interactions between the father and the infant. This diminished engagement leads to poor communication and stimulation [57], potentially causing long-term detrimental effects on the infant’s physical, cognitive, behavioral, and social development. Notably, fathers experience the highest rates of depression during 3 to 6 months postpartum [58]. Their critical role in a child’s development underscores the importance of addressing paternal PPD. 

The negative consequences of paternal PPD significantly impact partner relationships, infant bonding, and overall child development [55]. Paternal PPD has been associated with a range of later-life disorders in children, such as hyperactivity, anxiety, depression, attention deficit, oppositional defiant personality, and language delays [55]. In the short term, it can lead to infants exhibiting more challenging temperaments, including prolonged crying, sleep difficulties, and challenges in father-infant interactions. Breastfeeding infants may show poorer feeding behaviors and develop insecure attachments towards caregivers. In the medium term, children of fathers with PPD start to exhibit behavioral issues and emotional problems. Long-term consequences include a higher risk of lower psychosocial functioning, increased suicidal ideation, and a greater likelihood of depression [59].

Effect of COVID-19 on patients with paternal postpartum depression

While maternal postpartum depression has been widely researched, diagnosed, and promptly managed, little light has been thrown on paternal postpartum depression. The negative influence of COVID-19 on the psychological state of men and women has been widely established but the impact is far higher in the vulnerable population, including couples venturing into parenthood. After the advent of the global epidemic, the increasing prevalence of postnatal depression has been seen worldwide, and as a result, exacerbating mental health risks for new parents [60].

The prevalence of paternal postpartum depression escalated by 5.02% during the pandemic to reach,13.82% [61]. Before the transmission of COVID-19, a meta-analysis found that the occurrence rate of paternal postnatal depression within the initial year after childbirth was 8.8% [62]. Numerous factors can be attributed to increasing prevalence that can be categorized into: paternal, maternal, infant, pandemic-related stressors, interpersonal, and socioeconomic factors. Paternal factors attributed to an increase in depressive symptoms post-childbirth include a history of depression before conception of pregnancy [63-67] adverse childhood experiences [58], smoking, and intimate partner victimization violence [68]. Maternal factors negatively influencing their partners’ mental health comprise depression in postnatal mothers [10, 63] and previous unfavorable pregnancy outcomes [69]. Certain Infant factors such as preterm birth [67, 68] and poor infantile health [70] are linked with greater odds of paternal PPD.

Pandemic-related stressors can be positively attributed to a higher prevalence of PPD post-COVID-19. A narrative review conducted on PPD during COVID-19 found that the imposition of travel restrictions during the pandemic caused an increased risk of developing depressive symptoms [63]. A prospective birth cohort study and an online cross-sectional study conducted in Japan revealed that the fear of COVID-19 and an unstable economy during the pandemic [68] was associated with an increased likelihood of postnatal depression in fathers. Interpersonal factors playing a role in depressive symptoms in fathers during the pandemic can be attributed to participation in a paternal class based on a prospective cohort study [67], diminished marital content [68], poor family function [61], and unintended pregnancy [70]. Additionally, increased levels of life changes because of COVID-19 were linked to reduced levels of communication and dissatisfaction in couples. This observation aligns with research indicating that health worries among postpartum mothers and fathers, along with COVID-19-related restrictive measures, played a role in relationship challenges, therefore impacting their psychological well-being [71]. Socioeconomic facets amid the global epidemic such as low family resources [68], unemployment, low social support, and financial strain [10, 69, 70] have been consistent in studies ranging from meta-analysis to cross-sectional studies in influencing the postpartum mental health of men.

The International Marcé Society for perinatal mental health and the inclusion of fathers

The International Marcé Society is an international interdisciplinary organization that has the goal of holistic and inclusive perinatal care and has been guiding perinatal (prenatal and postpartum) health professionals for many years. The society operates to improve the mental health of the mother, baby, and father [72]. Members of the society are pediatricians, obstetricians, psychiatrists, nurses, early childhood specialists, psychologists, midwives, and other healthcare professionals [72]. By encouraging researchers and clinicians to become international members of their society, they can further decode the importance of fathers in child-rearing. There is a Special Interest Group (SIG) for fathers that enables social support for the father and ultimately the family, assesses the present state of paternal depression, and arranges webinars to disburse this information [73]. 

Fisher et al, in 2021, ask that the International Marcé Society place more focus on the inclusion of fathers in the conversation regarding perinatal research and mental health. Clinical training, a standardized policy, and research into interventions can all result in better support for paternal mental health in the perinatal period combined with the Marcé Society’s focus [74]. Marcé Society’s website has a compendium of knowledge related to scales and questionnaires, where to find care providers, child psychology webinars, and more. These resources enable the common layman to navigate difficult scenarios in early childhood [75]. Links to alternative support groups and care providers have also been populated [76, 77]. Dissemination of this information regarding the father/mother/infant dynamic and its potential stressors will be useful in reinforcing the ideal balanced parental dynamic.

Perinatal interventions for fathers suffering from paternal PPD

There are no specific treatment modalities for PPD in men despite its severity. Nonetheless, depression can be treated with a variety of evidence-based techniques in the fields of individual psychotherapy, couple psychotherapy, and pharmacological agents. These consist of cognitive behavioral therapy (CBT), behavioral couple therapy (BCT), and antidepressant medication [78].

Cognitive Behavioral Therapy (CBT)

The primary goal of CBT is to recognize erroneous and negative thoughts by highlighting the connection between emotions, ideas, and actions [79]. Cognitive behavioral therapy blends cognitive and behavioral therapies in a way that enables the patient to recognize unhelpful thought, emotional, or behavioral patterns and substituting them with constructive ones. CBT encompasses a range of strategies that include therapeutic approaches like cognitive therapy, dialectical behavior therapy (DBT), multimodal therapy [80], and rational emotive behavior therapy (REBT).

Behavioral Couple Therapy (BCT)

The phrase "behavioral couples therapy" refers to a broad category of couples therapies that make use of behavioral strategies like reinforcement that are founded in the concepts of operant conditioning [81]. BCT is appropriate for couples experiencing relationship difficulty due to depression or poor mood that affects one or both partners. These therapeutic procedures help with case creation, assessment, and teaching couples new techniques for resolving conflicts [81].

Psychosocial and Psychological Interventions

Psychosocial therapies are useful in helping parents who have experienced perinatal loss to deal with their sorrow, anxiety, and depression. Medical personnel are qualified to offer psychosocial treatment, which can be increased using technology [82]. Research supports the use of psychosocial treatments such as professional nondirective counseling and peer support to be effective in patients with mild PPD [83].

Pharmacological Intervention

Doctors tend to use pharmacological interventions as a last resort strictly adhering to the fact that monotherapy is preferable over polypharmacy. It is best to start antidepressant medication at a low dose and increase it gradually if needed [84]. The first-line antidepressant medications for PPD treatment are selective serotonin reuptake inhibitors (SSRIs). Examples include sertraline, paroxetine, and fluoxetine which are normally selected as first-line treatment options. With an evidence base comparable to that of sertraline and paroxetine, patients with moderate to severe PPD symptoms may benefit from the prescription of tricyclic antidepressants (TCAs), nortriptyline, and imipramine. Mild to moderate PPD is treated preliminary with psychotherapy [84]. Moderate to severe PPD is treated with psychotherapy and antidepressant medications as well [84]. A psychiatric emergency is considered in patients having PPD with suicidal/homicidal thoughts or in patients having PPD with psychotic features [84]. The recent FDA approval of brexanolone, an allopregnanolone-based treatment specifically developed for PPD, opens questions about the efficacy of the compound [85]. 

Another widely notable procedure in treatment is the use of video-feedback techniques. The audiovisual records of parent-child interactions are the most beneficial therapies during the prenatal period. Parents' sensitivity is enhanced by watching the films and receiving the therapist's advice, comments, and encouragement. This helps the parents take on a more suitable attitude toward the child and their spouse. An integrative approach of this kind fosters the mental and emotional health of the couple as well as the formation of strong attachment relationships, all of which have a favorable and long-lasting impact on the child's development. Such techniques include Systematic Training in Effective and Enjoyable Parenting (STEEP), Video-feedback Intervention to promote Positive Parenting (VIPP), Child Adult Relationship Experimental (CARE), and Lausanne Trilogue Play (LTP) video feedback [86].

Limitations in paternal PPD literature

Paternal PPD is a trending topic in research, but distorted study designs may create falsified skewed statistical evidence. Longitudinal studies are susceptible to high attrition rates, small sample sizes, and flawed correlations due to inaccurate follow-up periods that lead to estimation biases [87]. Prospective studies provide multiple sampling points with fixed interval follow-up appointment periods and should be implemented to counteract incorrect statistical results [88]. Cross-sectional designs and articles are subject to consensual bias and may not explain causality creating susceptibility to research knowledge gaps [89]. Publication bias must be considered since only English-speaking studies are referenced, negating cross-cultural data that would give us more comprehensive and accurate results. Sample bias includes the underrepresentation of low and middle-income countries. Meta-analysis studies are subject to confounding variables as they cannot explain causality allegorical to cross-sectional studies [10].

Imprecise measures of depression include dismissing comorbid health conditions. Some depression scales, i.e., the EPDS, present flaws by overlooking some fathers with depression since there is a low specificity and low positive predictive value (PPV) [90]. Pre-pregnancy marital status and comorbid health conditions (i.e., anxiety or other psychological health conditions) may contribute to paternal PPD. We cannot solely conclude accurate findings by looking merely at depression or doing combined analysis related to spousal reports. Berkson bias can occur if we consider men who had depression before their partner's pregnancy when examining postpartum depression [91].

Breastfeeding and oxytocin-prolactin cycles may contribute to decreased paternal-child bonding leading to paternal PPD [92]. Biological risk factors may vary between each individual and further studies are needed to show whether chemical imbalances lead to paternal PPD. Various researchers suggest that decreased testosterone levels correlate to lower aggression, better concentration in parenting, and stronger attachment with the infant. The estrogen level in men begins to increase during the last month of their partner's pregnancy until the early postpartum period. Lower levels of cortisol among men may be related to difficulties in father-infant bonding and associated depressed mood. Low vasopressin levels, which increase after the birth of the child in a way analogous to the oxytocin level of the mother, contribute to paternal PPD. Brain imaging of expectant fathers can evolve our understanding of neurohormonal pathways and their role in paternal PPD [12]. 

Recent ecological studies portray fathers playing a more significant role in parenting, yet they did not learn appropriate parenting skills from their fathers or other male role models. These fatherhood skills include contributing to household chores and using active and physical involvement to create emotional connections with newborns. High-quality father involvement during childhood promotes higher employment rates, healthier relationships, and other favorable life qualities for the child well into adulthood [93]. The discrepancies in gender roles might cause anxiety, especially for first-time fathers, and lead to a greater risk of paternal PPD. Cultural factors stemming from collective societies including over-involvement of in-laws, especially mothers-in-law, can influence fathers' involvement in parenting. Community-based educational workshops help fathers understand their expected roles and alleviate the hesitance of fatherhood. Artificial insemination must be considered as a factor that may lead to paternal PPD. Lifestyle factors in male reproductive health have contributed to a global decline in semen fertility over recent decades. These factors include smoking cigarettes, alcohol intake, use of illicit drugs, obesity, psychological stress, advanced paternal age, dietary practices, and coffee consumption [94]. Paternal emotional support needs to be considered before, during, and after the post-pregnancy period. Men will express their depression as anger and anxiousness to mask sadness. Cohort studies for paternal depression may be misleading as it does not consider the SIGECAPS (sleep, interest, guilt, energy, concentration, appetite, psychomotor, and suicide) scale based on DSM-5 [95, 96].

Recommendations for improving outcomes for paternal PPD

The arrival of a new baby is a huge transformative moment in the family’s life. The birth of the child can bring about a lot of emotional, and physical changes and/or challenges in both parents. While maternal blues and postpartum depression have a standard set of treatment plans, paternal postpartum depression is very often understudied and underdiagnosed [12].

Meta-analyses of PPD illustrate that approximately 10% of fathers will experience a depressive episode during the perinatal period [97]. Because of the prevalence and impact of this disorder, it is imperative to identify and offer treatments and interventions designed specifically for this population [97]. Screening for depression in men during the first year postpartum should be considered in the regular postpartum checkups [31]. To begin with, it is very important to acknowledge the issue and the problems faced by the father. Educational programs help the father and mother understand what they are supposed to expect and be prepared for. Apart from being understudied and underdiagnosed, paternal is largely unheard of [55]. For the same reasons, an educational program for PPD in both fathers and mothers would be more effective (as opposed to only maternal PPD educational programs). Paternal PPD is closely related to the mother and her mood, and emotions, and vice versa. And hence, a combined educational program would have more benefits [51, 98, 99].

The most effective support system comes primarily from the partner. Being prepared and having open, clear communication among the couple may ease the father's stress. Secondly, society should be more open and supportive of the new father. For instance, they paid paternity leave or parental leave. There are 45 countries with paid paternity leave concepts. These countries have better mental health stability in the father. Sweden has a shared leave policy with a starting pay of 80%. It incorporates equal involvement of both parents and neonates and improves emotional connection. To add to the benefits, the father's long presence in the neonates shows better developmental outcomes in the child [100]. Additionally, psychological interventions such as CBT, interpersonal therapy, and group sessions are very effective and helpful for the parent's emotional and mental well-being [51, 99].

Due to its feasibility and benefits, skin-to-skin can be considered as prevention for paternal PPD and as the first-line non-pharmacological treatment for PPD [100]. Skin-to-skin bonding with the neonate has shown benefits with maternal postpartum depression but has not been investigated extensively in fathers. A population-based cohort project (UPPSAT) in Uppsala, Sweden used the EPDS at 6 weeks and 6 months postpartum and the Postpartum Bonding Questionnaire at 6 months postpartum. 727 couples participated in the above study. Depressive symptoms at 6 weeks postpartum are associated with impaired bonding with the infant at 6 months postpartum for both mothers and fathers [101, 102]. Certain studies show the skin-to-skin approach to be an effective way to emotionally connect the dad and the neonates. It has a positive effect on the development of the neonate as known, however, it also shows positive mood elevation and a happy state of mind in the dad. Since the symptoms and mechanism remain the same as maternal PPD, the pharmacotherapy will remain the same. The recommended first-line treatment is SSRI, such as sertraline.

## Conclusions

Our article studies PPD and its ramifications in the prenatal, perinatal, and postpartum periods. Global incidence and prevalence of paternal depression are related to occupational, genetic, and spousal anomalies. Paternal emotional satisfaction is directly proportional to paternal-child bonding. This eliminates the research gap that maternal emotions are solely experienced during pregnancy. Though CBT recognizes erroneous and negative thoughts by highlighting the connection between emotions, ideas, and actions, further studies on community-based educational workshops and groundbreaking epidemiological studies (Dialectical Behavioural Therapy) can teach parents how to regulate emotions during pregnancy and post-pregnancy and these concepts can be applied cross-culturally. This will help incorporate the behavioral changes and extinguish the Hawthorne effect involved with questionnaire studies, which are currently the predominant research method.
